# Coupled Femtoexcitons, Free Carriers, and Light

**DOI:** 10.1021/acs.nanolett.5c01871

**Published:** 2025-08-06

**Authors:** D. Gill, S. Shallcross, W. Chen, J. K. Dewhurst, S. Sharma

**Affiliations:** † Max-Born-Institute for Non-linear Optics and Short Pulse Spectroscopy, Max-Born Strasse 2A, 12489 Berlin, Germany; ‡ 28286 Max-Planck-Institut fur Mikrostrukturphysik Weinberg 2, D-06120 Halle, Germany; ¶ Max-Planck-Institut fur Mikrostrukturphysik Weinberg 2, D-06120 Halle, Germany; § Theoretical Solid-State Physics, Freie Universität Berlin, Arnimallee 14, 14195 Berlin, Germany

**Keywords:** ultrafast, exciton dynamics, free carrier, dynamics, exciton dissociation

## Abstract

Nonequilibrium quantum matter generated by ultrafast laser light opens new pathways in fundamental condensed matter physics, as well as offering rich control possibilities in “tailoring matter by light”. Here we explore the coupling between free carriers and excitons mediated by femtosecond-scale laser pulses. Employing monolayer WSe_2_ and an *ab initio* treatment of pump–probe spectroscopy, we find that, counterintuitively, laser light resonant with the exciton can generate massive enhancement of the early-time free-carrier population. This exhibits a complex dynamical correlation to the excitons, with an oscillatory coupling between the free carrier population and exciton peak height that persists. Our results both unveil “femtoexcitons” as possessing rich femtosecond dynamics as well as, we argue, allow tailoring of early-time light–matter interaction via laser pulse design to control simultaneously excitonic and free-carrier physics at ultrafast times.

## Graphical abstract

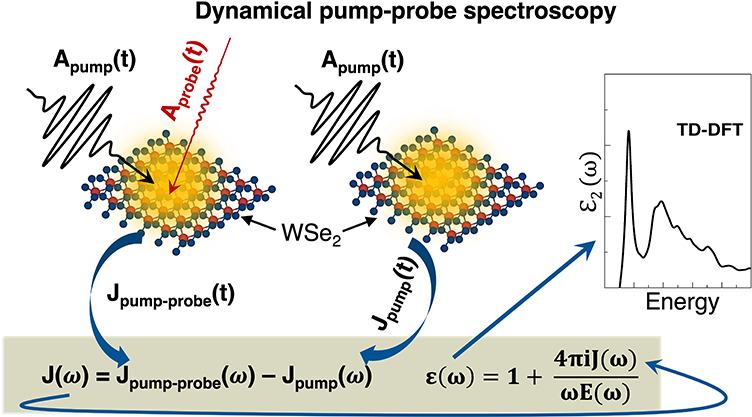

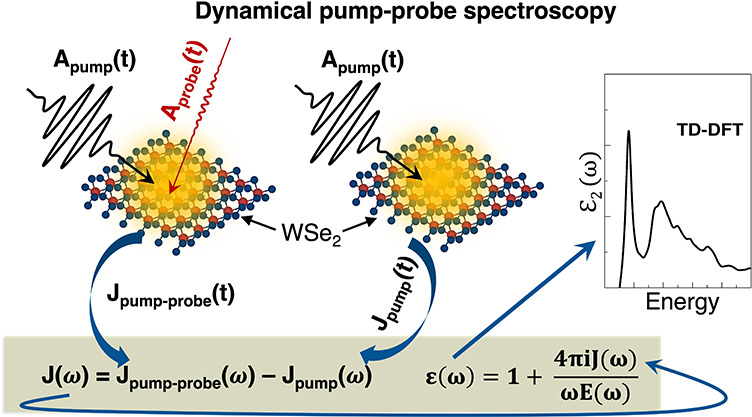

Traditionally light has been used as a probe for material properties; however, in the present era of atto- and femtosecond lasers, the role of light has become 2-fold. Pump laser light generates a nonequilibrium state of matter, and probe laser light then explores the nature of this new quantum state of matter.^1–8^ This has led to the emergence of new fields of physics such as femtomagnetism,^9,10^ lightwave control over spintronics,^11,12^ and ultrafast valleytronics,^13^ to name but a few examples. Pump laser photons impart their energy to matter, creating an out-of-equilibrium quantum state, the probing of which is highly complex; photons (of pump as well as probe light), electrons, and nuclei *dynamically couple* with each other both during and after excitation. For example, light interacts with spins via spin–orbit coupling, which is, in principle, a material-specific property. However, the pump light pulse generates a nonequilibrium state of excited charge, and these excited electrons act as a new state of matter and hence feel a spin–orbit coupling different from the original material, making the coupling dynamical^14–16^ and hence also a property of the light and not just the material.

In semiconductors, one such quasi-particle that is created and probed by light is the exciton; light induces electrons to excite to the conduction band (CB), leaving behind a hole, and this hole and the excited electron then form a bound electron–hole pair called an exciton. Such excitons form a localized band in the gap, dramatically changing the electronic structure of the material. Probing the dynamics of such excitons upon light-wave pumping requires the treatment of pump light, electrons, excitons, and probe light, all on the same footing. A method capable of doing so at present is the Kohn–Sham–Proca (KSP)^17^ approach to time-dependent density functional theory (TD-DFT),^18^ which is a fully *ab initio* pump–probe spectroscopic approach.

In the present work, using KSP, we unravel the complex light-wave-exciton dynamics in a monolayer (ML) of transition-metal dichalcogenide (TMDC), WSe_2_. We pump the material and probe it at various time delays, allowing both pump and probe pulses to transiently create and destroy excitons, which also dynamically interact with free carriers. We show that this dynamics is highly complex; counterintuitively, pumping the material at the exciton energy level leads to a dramatically enhanced creation of free carriers in the CB compared to pumping these free carriers directly into the CB. The excitonic response further shows an intricate interplay of interacting excitons and free carriers, mediated by the pump pulse, leading to persistent oscillations in the free-carrier density, in step with the exciton binding energy and intensity.

In order to unravel the complex interplay of light, excitons, and fermions, we study the dynamics of these in an example system^20^ of a ML-WSe_2_. We drive this TMDC out of equilibrium by selective laser pumping in two different ways: (a) resonant pumping—the material is pumped with laser light of central frequency equal to the excitonic frequency (1.67 eV), leading to exciton creation—and (b) off-resonant pumping—here the material is pumped with laser light of central frequency 3.0 eV. Because ML-WSe_2_ is an insulator with a direct gap of 2.2 eV (an indirect gap of 2.12 eV)^21^ and an exciton binding energy of 0.40 eV,^22^ a resonant pump pulse does not allow for any significant direct creation of excited charge in the CB, i.e., free carriers. In contrast, off-resonant pumping allows for the direct creation of free carriers, some of which will form excitons. In order to ensure (a) and (b), the pump pulse is chosen to have fluence (1.70 mJ/cm^2^), which allows significant excitation while remaining in the regime in which single-photon processes dominate and a duration (25 fs) such that the selected central frequency dominates (for the frequency spectrum of the pulses, see Figure 1 of the Supporting Information, SI). Following the excitation with these two types of pulses, we then explore the dynamics of free carriers as well as excitons. Our theoretical method of choice is the fully *ab initio* KSP method^17,23^ within TD-DFT (for details of the method and computations, see the section about the KSP equation and computational details in the SI, respectively). This method is capable of treating the dynamics of excitons and free carriers^24,25^ on the same footing, while they both interact with the laser pump and probe pulses.

*Free-carrier dynamics*: Using the KSP scheme, we calculate the static excitonic spectra^23^ of WSe_2_ (Figure 1a); the exciton peak in the response function calculated using KSP (red curve) agrees well with the experimental (black curve) absorption spectra;^19^ in close agreement with experiments, we find the exciton binding energy to be 0.44 eV. As expected, excitons are missing from the random-phase approximation (RPA) absorption spectrum, i.e., when the TD-DFT KS equations are solved in the absence of the Proca equation (blue curve). Having captured the correct static response of the material to light, we now follow the dynamics of the material upon resonant and off-resonant light-wave pumping. The dynamics of free carriers (the number of electrons in the CB) is shown in Figure 1b; in striking contrast to off-resonant pumping (green), for the case of resonant pumping (red), there is an enhancement in the free-carrier density (by a factor of ∼10). There can be only two causes of such free-carrier excitation with pump light at a subgap frequency: (i) two photon processes or (ii) the creation of free carriers via light-wave interaction with excitons. The former can be ruled out by inspection of the excited charge density in the absence of excitons, and as can be seen in Figure 1b, in this case (blue), the excited charge falls to zero. From these results, the picture that emerges is that of a resonant laser pulse creating excitons and dynamically interacting with these excitons to generate extra free carriers (via radiative dissociation of excitons).

**Figure 1:**
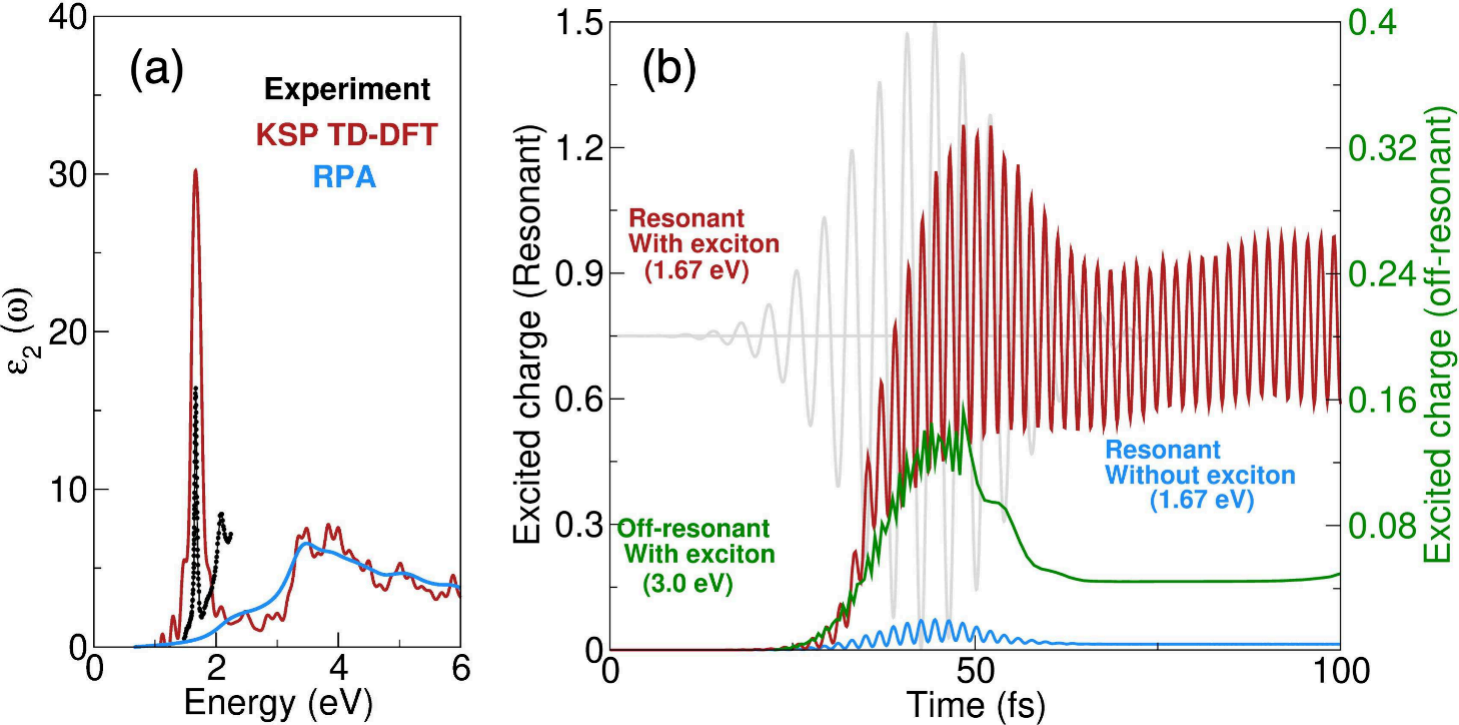
Influence of exciton dynamics on free carriers. (a) Absorption spectra in the presence and absence of excitons. For comparison, experimental data^19^ are also shown. (b) Dynamics of the excited charge in the presence (red) and absence (blue) of excitons for resonant (at ω = 1.67 eV) and in the presence of exciton dynamics for off-resonant (at ω = 3.0 eV) pumping (green). The vector potential of the resonant pump pulse is shown in gray. Resonant pumping strongly couples photons, excitons, and free carriers to (i) dramatically enhance the free-carrier population and (ii) generate a persistent oscillatory coupling between the exciton and free-carrier populations. Note that the experimental data shown in panel (a) are adapted with permission from ref 19. Copyright 2018 American Physical Society.

*Exciton dynamics*: We now follow the dynamics of these excitons (Figure 2), and for this, we will use the pump–probe spectroscopic method, wherein we probe the excitonic response of ML-WSe_2_ at various time delays after pumping, as would be done in any potential spectroscopic experiment (for details and a schematic of this, see Figure 4). Upon resonant pumping, excitons precede free carriers; by probing ML-WSe_2_ during the rising edge of the pump pulse and before excitation of free carriers (first dotted lines in Figure 2a), we see an excitonic peak in the response, indicating exciton formation. However, as the pump pulse reaches its maximum amplitude, there is significant bleaching of the excitonic response, that is, a decrease in the excitonic peak intensity (Figure 2c). This decrease in the peak intensity is an indicator of the dissociation of excitons, and this happens at the same time as there is an increase in the free carrier density, further cementing the facts that the exciton dissociation is radiative and that excitons and free carriers show a correlated dynamics upon ultrafast laser pumping.

**Figure 2:**
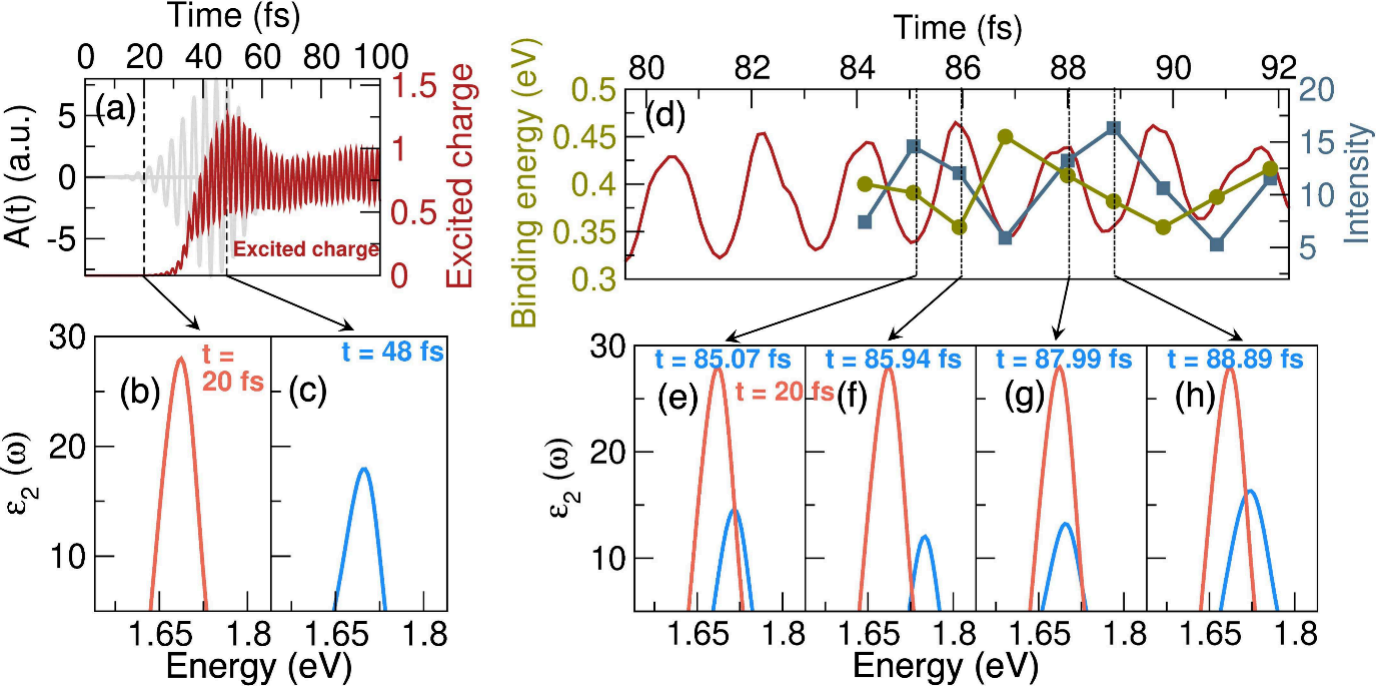
Strong coupling of photons, electrons, and excitons. (a) Dynamics of resonantly pumped free carriers (red) in the CB (the vector potential of the pump pulse is shown in gray; see computational details in the SI for pulse parameters). Dotted black lines indicate the times at which the material is probed, and the corresponding absorption spectra are shown in panels b and c. (d) Dynamics of the exciton binding energy (green) and peak intensity (blue), together with the dynamics of excited free carriers (red) in a time window of 80–90 fs. Dotted black lines indicate the times at which the material is probed, and the corresponding absorption spectra are shown in panels e–h.

This general bleaching of the excitonic peak after light-wave pumping is observed in previous experiments for various materials.^4,6,26–29^ A closer inspection reveals that the dynamics of the excitonic peak intensity (during and after pumping) is more complex than steady bleaching. By probing the material at various time delays, we find that the excitonic peak intensity as a function of time is oscillatory (blue line in Figure 2d); this is an indicator of the dynamical formation and destruction of excitons under the impact of a pump pulse (each blue and green point in Figure 2d requires a numerically extensive calculation procedure, as outlined in Figure 4, and hence these data points are calculated at a few selected time steps). Such an oscillatory response of the excitonic peak intensity due to the dynamics of excitons has recently been seen in experiments: in ref 30, the coupling of two excitons produces early time oscillations in the absorption, while in ref 31, coupling to a vibronic state generates oscillations on a longer (hundreds of femtoseconds) time scale. The ultrafast time scale of the oscillation seen in this work implies a mechanism involving only electrons and light. In all of these cases, a “two-state system” arises from coupling an exciton to a second degree of freedom: a second excitonic peak in the case of ref 30, a vibronic state in the case of ref 31, and free carriers in the present work.

To explore this, we note that for most of the time delays the reduction in the exciton peak intensity is correlated with an increase in the free carrier density (red and blue lines in Figure 2d and absorption spectra in Figure 2e–h) and vice versa. A similar process was experimentally unveiled in carbon nanotubes, where a decrease in the excitonic peak height was correlated with an increase in the population of a second excitonic state,^32^ instead of the CB, as in the present case. The binding energy of the excitons also shows in-step oscillations (see the green curve in Figure 2d), indicating the role of screening in exciton^27^ dissociation. We note that the changes in the KS band gap^4,33–35^ during this early-time dynamics are very small. A picture that now emerges is thus of a dynamical system of correlated excitons, free carriers, and light: (1) upon resonant pumping, excitons are first created; (2) then upon interaction with the pump laser pulse and with each other, some of these excitons dissociate radiatively; (3) this leads to an increase in the free carrier density; some of these free carriers contribute toward the screening of excitons, while at the same time, some of these free carriers form new excitons (for the schematic, see Figure 3a). Given that our system is isolated and decoupled from phonons (and any other energy loss mechanism), the cycle of excitation and de-excitation of free carriers continues.

**Figure 3:**
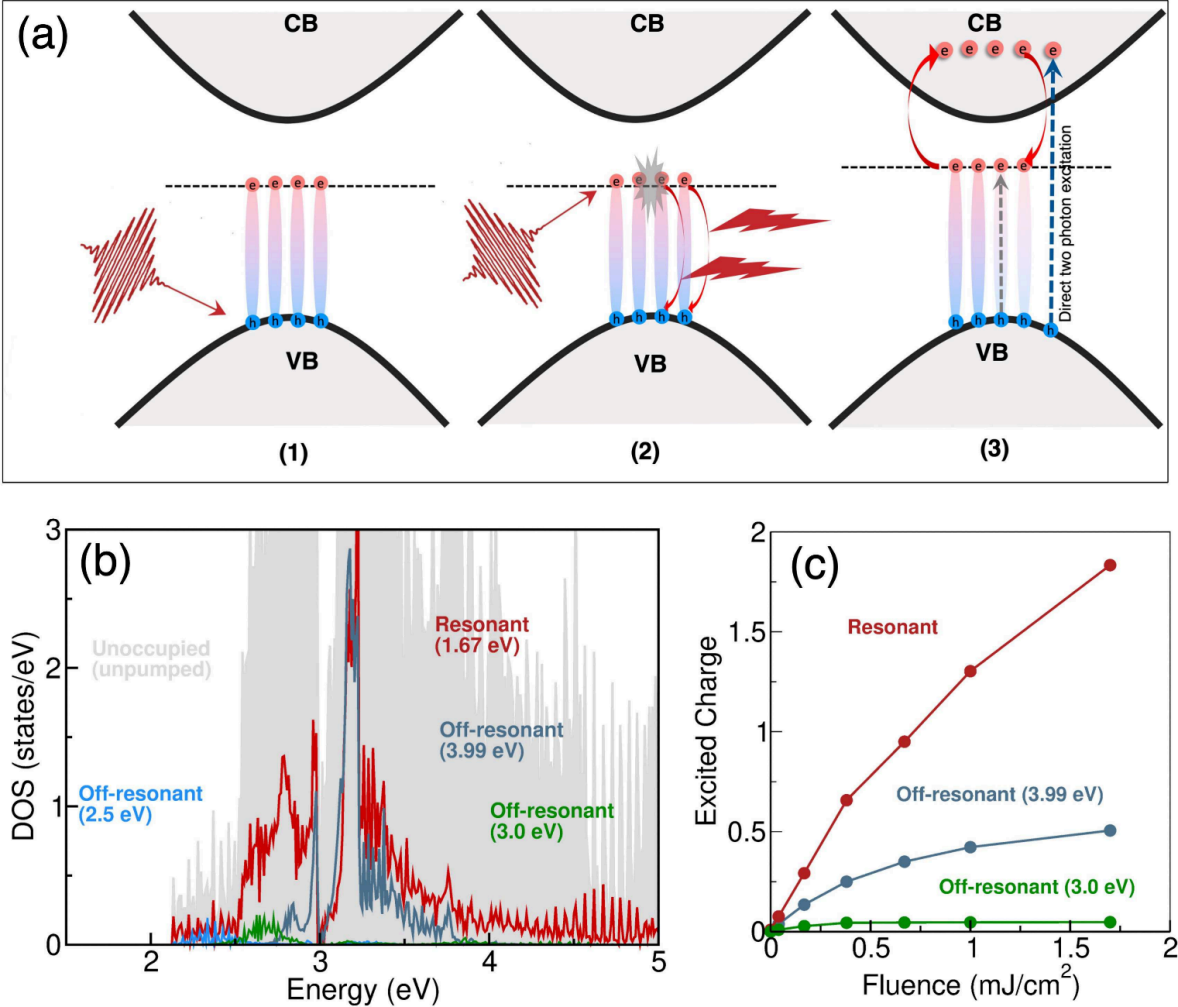
(a) Schematic illustration of exciton-mediated free-carrier excitation: (1) the laser pump creates excitons by resonant excitation; (2) exciton–light and exciton–exciton interaction drives exciton recombination and photon emission; (3) these photons then lead to excitation of the charge into the CB. (b) Unoccupied ground-state DOS, illustrated in gray, revealing a low density of available states at the CB edge with a much increased density of available states at higher energies within the CB. For off-resonant pumping, direct excitation to the CB edge quickly saturates due to Pauli blocking, with the transient DOS of the excited charge shown in light blue, green, and dark blue for three different frequencies of the pump pulse. In contrast, resonant pumping excites particles into a region with a high density of available states, facilitating significant generation of free carriers (shown in red). (c) Excited charge as a function of the fluence for resonant (red) and off-resonant (green and blue) pumping. In the former case, an increase with the fluence is observed, while in the latter cases, saturation occurs at very low fluence.

These results show remarkable similarities with earlier experimental data; a radiative dissociation of excitons due to resonant pumping has previously been observed.^36,37^ This dissociation becomes pronounced as the pump pulse peaks^38^ and is accompanied by an increase in the free-carrier population.

*Availability of states and pulse frequency*: From these results, it is clear that free carriers and excitons show early-time-correlated dynamics mediated by the pump laser pulse. We can further investigate the underlying microscopic physics of this dynamics, and the role of pulse frequency, by inspecting the transient electronic structure of ML-WSe_2_. Upon resonant pumping, free carriers are generated via a two-step process of (i) dissociation and, subsequently, (ii) excitation of an electron to the CB. This generates both a bleaching of the excitonic peak (Figure 2) and a transient free-carrier population at energies *e*_*x*_ + ℏω to *e*_*x*_ + 2ℏω, where *e*_*x*_ = 1.67 eV is the excitonic state energy and ω is the photon energy. This corresponds to one- and two-photon excitations after dissociation (Figure 3b, red curve). Off-resonant pumping differs dramatically. This can be seen in Figure 3b, in which we display the excited free carriers for three excitation energies: ℏω = 2.5 eV (light blue), ℏω = 3.0 eV (green), and ℏω = 4.0 eV (dark blue). In each case, the excited free carriers are found to be close to the energy ℏω, demonstrating the dominance of one-phonon excitations for this case. This difference illustrates the very different underlying physical processes: excitation from a many-body state at resonant pumping and free-carrier excitation across the gap in the case of off-resonant excitation. Note that in both cases the transient spectrum is “shaped” by the availability of states determined by the ground-state spectrum. (The results for several other frequencies can be found in Figure 2 of the SI.)

*Laser pulse fluence*: These results highlight the importance of pump pulse parameters, such as the central frequency, in mediating free-carrier-exciton-correlated dynamics. In this result, both the pulse duration and fluence (1.7 mJ/cm^2^) were held fixed, and we now consider the fluence dependence of the light-induced free-carrier-exciton dynamics (the results for varying the duration of the pulse can be found in Figures 3 and 4 of the SI). At off-resonant pumping, the excited charge saturates with increasing fluence, at a point determined by the availability of states at the excitation frequency: in Figure 3c, we see that 3.0 eV pumping (for low availability of states, see panel b) saturates at considerably lower fluences than those for 4.0 eV pumping (higher availability of states). The saturation for the off-resonant pumping is therefore due to Pauli blocking.^39–41^ Resonant pumping, because excitation from the correlated exciton state allows both one- and two-photon excitation, has a significantly increased availability of states to excite into. The fluence dependence in this case is thus quite different, and slowing in the rate of radiative exciton dissociation^42^ is the leading cause for the weak nonlinearity seen in the excited charge. Such breaking or recombination of excitons after pumping and an associated linear dependence on the pump fluence (increased dissociation with fluence) has been experimentally observed.^42–45^ These works, which focus on later picosecond times in the regime of excitonic scattering and relaxation, have also revealed a complex intertwined dynamics of excitons, electrons, and holes. In the present work, by studying subcycle dynamics, we show that such complex correlated dynamics occurs even at early femtosecond times in which there is correlation also with the pump light itself.

At the picosecond times at which a laser excited state returns to equilibrium, recent experiments report coupling of excitons, free carriers, and lattice excitations. Here we have found that, already in the subcycle structure of the laser pulse, excitons and free carriers are intimately coupled. This underpins a very rich early-time dynamics in which the populations of free carriers and excitons exhibit a strong dependence on each other. A notably counterintuitive example of this is that laser pumping resonant with the exciton generates a free-carrier population significantly greater than that obtained by laser pumping directly into the CB, an effect driven by light-induced dissociation of excitons.

This fundamentally intertwined nature of light, free carriers, and excitons in femtosecond nonequilibrium dynamics is dramatically illustrated by an in-step oscillation of free-carrier and exciton populations and should be observable in experiments with good time resolution.

Our results thus point toward a rich control over exciton dynamics via free-carrier excitations and, vice versa, upon free carriers via exciton pumping mediated by the laser pump pulse. Furthermore, because the availability of excited states plays a crucial role in the density of free carriers as well as excitonic properties (such as binding energy and intensity), laser pulse design and manipulation of the density of states (DOS), both now raised to art forms in modern condensed matter research, promise rich possibilities for tailoring nonequilibrium excitonic states by light, i.e., femtoexcitonics.

*KSP method*: TD-DFT^18,46^ rigorously maps the computationally intractable problem of interacting electrons to a Kohn–Sham (KS) system of noninteracting electrons in an effective potential. The time-dependent KS equation is

1 $$i\;\frac{\partial \psi_{j}\left(r,t\right)}{\partial t}=\left\{\frac{1}{2}{\left[-i\nabla -\frac{1}{c}\left[A\left(t\right)+A_{\mathrm{XC}}\left(t\right)\right]\right]}^{2}+v_{s}\left(r,t\right)\right\}\psi_{j}\left(r,t\right)$$

where ψ_*j*_ are the KS orbital and the effective KS potential *v*_s_(**r**,*t*) = *v*(**r**,*t*) + *v*_H_(**r**,*t*) + *v*_XC_(**r**,*t*) consists of the external potential *v*, the classical electrostatic Hartree potential *v*_H_, and the exchange-correlation (XC) potential *v*_XC_. The vector potential **A**(*t*) represents the applied laser field within the dipole approximation (i.e., the spatial dependence of the vector potential is absent) and **A**_xc_(*t*) the XC vector potential. This is generated by coupling of the KS equation ([Disp-formula eq1]) with the Proca equation:

2 $$a_{2}\;\frac{\partial^{2}}{\partial t^{2}}\;A_{\mathrm{XC}}\left(t\right)+a_{0}A_{\mathrm{XC}}\left(t\right)=4\pi J\left(t\right)$$

Here the gauge invariant current, **J**, is obtained by integrating the microscopic current density **j**, which is given by

3 $$j\left(r,t\right)=Im\overset{occ}{\underset{j}{\sum }}\psi_{j}\left(r,t\right)\;\nabla \psi_{j}\left(r,t\right)-\frac{1}{c}\left[A\left(t\right)+A_{\mathrm{XC}}\left(t\right)\right]\rho \left(r,t\right)$$

Thus, eqs 1 and 2 are coupled and simultaneously solved (for further details of this method, see ref 17).

The transient excitonic response is determined using the pump–probe method, as illustrated in Figure 4. In this approach, the system is pumped with a laser pulse [with vector potential **A**_ext_(*t*) = **A**_pump_(*t*)] and then probed, after a time delay of Δ*t*, with a very weak probe pulse [with vector potential **A**_probe_(*t*)]. This yields two kinds of currents: **J**_pump_(*t*) and **J**_pump–probe_(*t*,Δ*t*). The difference between the Fourier transform of these currents [**J**(ω,Δ*t*) = **J**_pump–probe_(ω,Δ*t*) – **J**_pump_(ω)] is then used to generate the response function:

4 $$\varepsilon \left(\omega ,\Delta t\right)=\frac{4\pi iJ\left(\omega ,\Delta t\right)}{\omega E\left(\omega \right)}$$

where *ε*(ω,Δ*t*) in the dielectric tensor and **E**(ω) is the Fourier transform of the electric field of the probe laser pulse, **E**(*t*) = −1/**c** δ**A**_probe_(*t*)/δ*t*.

**Figure 4:**
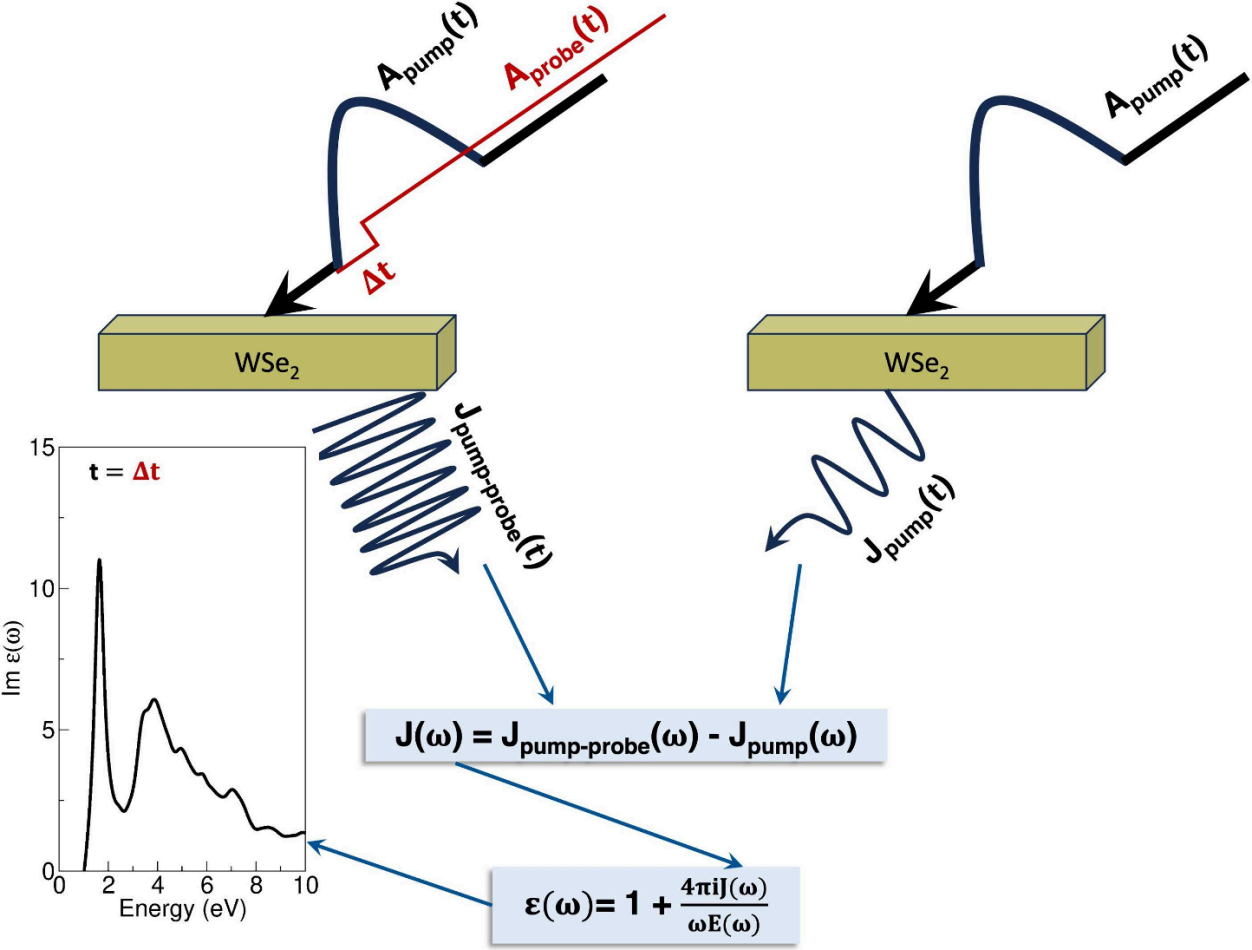
Schematic illustrating calculation of the transient excitonic response using the pump–probe approach. The charge current in the frequency domain **J**(ω), which is obtained by taking the difference of the current **J**_pump–probe_(ω) (current obtained when the system is exposed to a pump–probe pulse) and **J**_pump_(ω) (the current obtained when the system is exposed to a pump pulse), is used to calculate the dielectric function containing information on the excitonic physics at time *t* = Δ*t*.

It should be noted that while TD-DFT with the exact *v*_XC_(**r**,*t*), in principle, solves the many-body time-dependent problem, the treatment of excitons within periodic boundary conditions cannot be captured in this way. As discussed in recent works,^17,47^ the formal solution to this is to invoke an XC vector potential **A**_XC_(*t*), exactly as done in the present work via the “procedural functional” route represented by coupling the TD-DFT KS equation, eq 1, to the Proca equation, eq 2.

This coupling, importantly, implies a coupling of free carriers to excitons that is crucial in the description of excitonic physics out of equilibrium. To see this, we note that, from an initial condition **A**_XC_ = 0 at *t* = 0, a pump laser pulse implies that **A**_XC_(*t*) evolves to a finite value. This happens because a pump laser will generate current and, via the Proca equation ([Disp-formula eq2]), such a current implies a nonzero **A**_XC_(*t*). Because **A**_XC_(*t*) forms part of the vector potential of the TD-DFT KS equation, this immediately makes clear that the time-dependent KS wave function ψ­(**r**,*t*) has a functional dependence on **A**_XC_(*t*), thus coupling the free-carrier and excitonic systems.

## Supplementary Material



## Data Availability

All data involved in the production of the manuscript is available upon reasonable request.

## Notes

Computational details, frequency spectra of the pump pulses, excited DOSs at different off-resonantly pumped frequencies, free-carrier dynamics upon pumping with short-duration pulses, and Fourier analysis of the oscillations (PDF)
