# Supplementary material for: Coupled Femtoexcitons, Free Carriers, and Light
Source: Nano Lett. 2025 Aug 6;25(33):12439–45. doi: 10.1021/acs.nanolett.5c01871 (PMC12371882; doi:10.1021/acs.nanolett.5c01871)
Supplement: Supplementary file 1 [file nl5c01871_si_001.pdf]

Supplemental Information: Coupled femto-excitons, free carriers and light

D. Gill,[†] S. Shallcross,[†] W. Chen,[‡] J. K. Dewhurst,[*,¶] and S. Sharma[*,§]

†*Max-Born-Institute for Non-linear Optics and Short Pulse Spectroscopy, Max-Born Strasse 2A, 12489 Berlin, Germany*

‡*Max-Planck-Institut fur Mikrostrukturphysik Weinberg 2, D-06120 Halle, Germany*

Max-Born-Institute for Non-linear Optics and Short Pulse Spectroscopy, Max-Born Strasse 2A, 12489 Berlin, Germany

¶*Max-Planck-Institut fur Mikrostrukturphysik Weinberg 2, D-06120 Halle, Germany*

§*Max-Born-Institute for Non-linear Optics and Short Pulse Spectroscopy, Max-Born Strasse 2A, 12489 Berlin, Germany*

Institute for theoretical solid-state physics, Freie Universität Berlin, Arnimallee 14, 14195 Berlin, Germany

E-mail: dewhurst@mpi-halle.mpg.de; sharma@mbi-berlin.de

Computational details

In our work, we used the adiabatic local density approximation for the XC potential and all calculations were performed using the highly accurate full potential linearized augmented-plane-wave method, as implemented in the ELK code (version elk-10.3.12).[1] The ML-WSe_2 was modelled using a hexagonal unit cell, with in-plane lattice vectors:[2] a = [1.658, -2.869, 0] Å; b = [1.658, 2.868, 0] Å. A vacuum of 19.16 Å was used along the c-axis to emulate a mono-layer. All states upto an energy cut-off of 70 eV above the Fermi energy were used. A

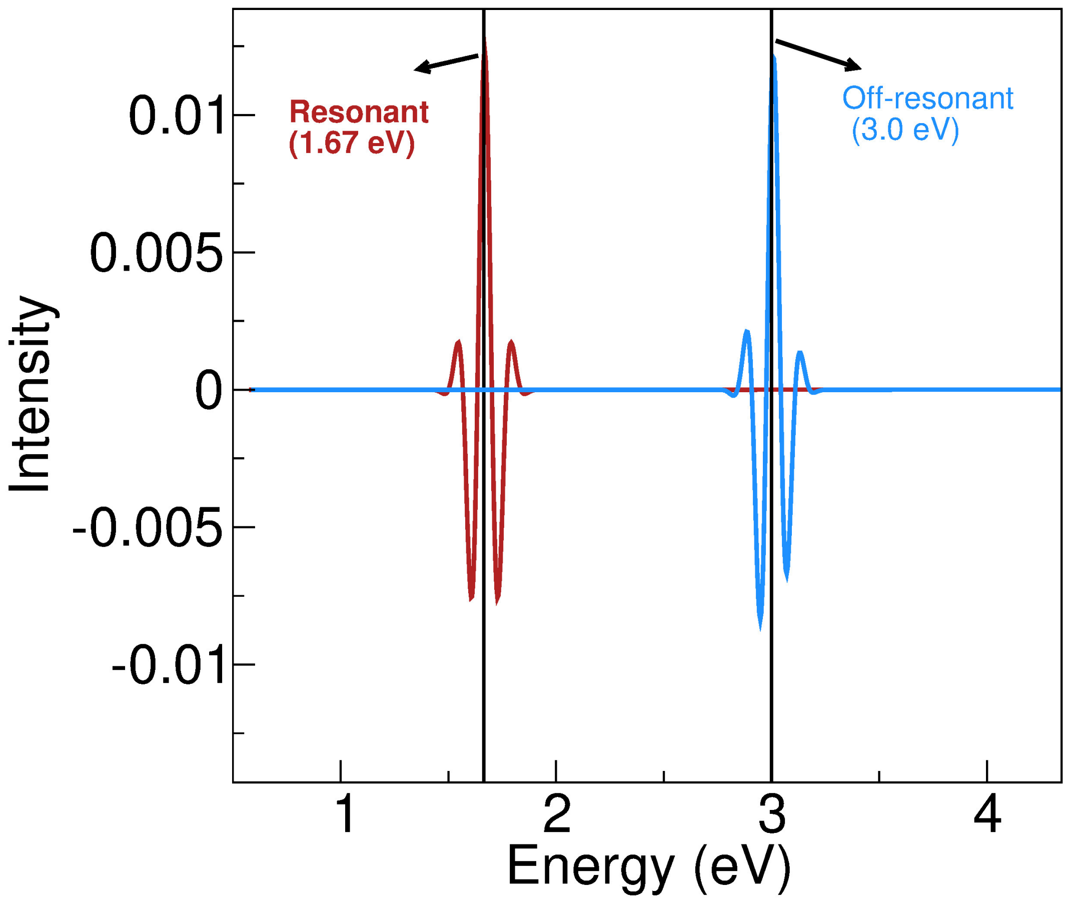

Figure 1: *Frequency spectrum of the pump pulses;* Fourier Transform of the vector potential of resonant and off-resonant pump laser pulses. Resonant pulse has frequency components around exciton frequency of 1.67 eV and off-resonant pulse around the gap energy of 3.0 eV. Most importantly, frequency spectrum of the two does not overlap.

k-point grid of 20x20x1 and a smearing width of 0.027 eV (313 K) was used. For the time propagation algorithm, a time step of .0012 fs and a total time of 120 fs was used (for details of time-propagation algorithm see Ref.[3]). The parameters for the Proca equation (Eq. 2 of the paper) were determined[4] to be $a_0 = 0.2$ and $a_2 = 100$. The parameters used for the off-resonant (and resonant) pulse are as follows: Fluence = 1.7mJ/cm^2; duration = 23.95 fs; frequency of 2.12 eV (1.67 eV). We note that the fluences quoted in the main manuscript and this Supplemental document represent the incident fluence of the pump pulse. In theory a very small part of this is absorbed (less than 10%) to cause electronic excitation.[5] A scissors correction of 0.585 eV was used for shifting the KS band-gap to match experiment.[6]

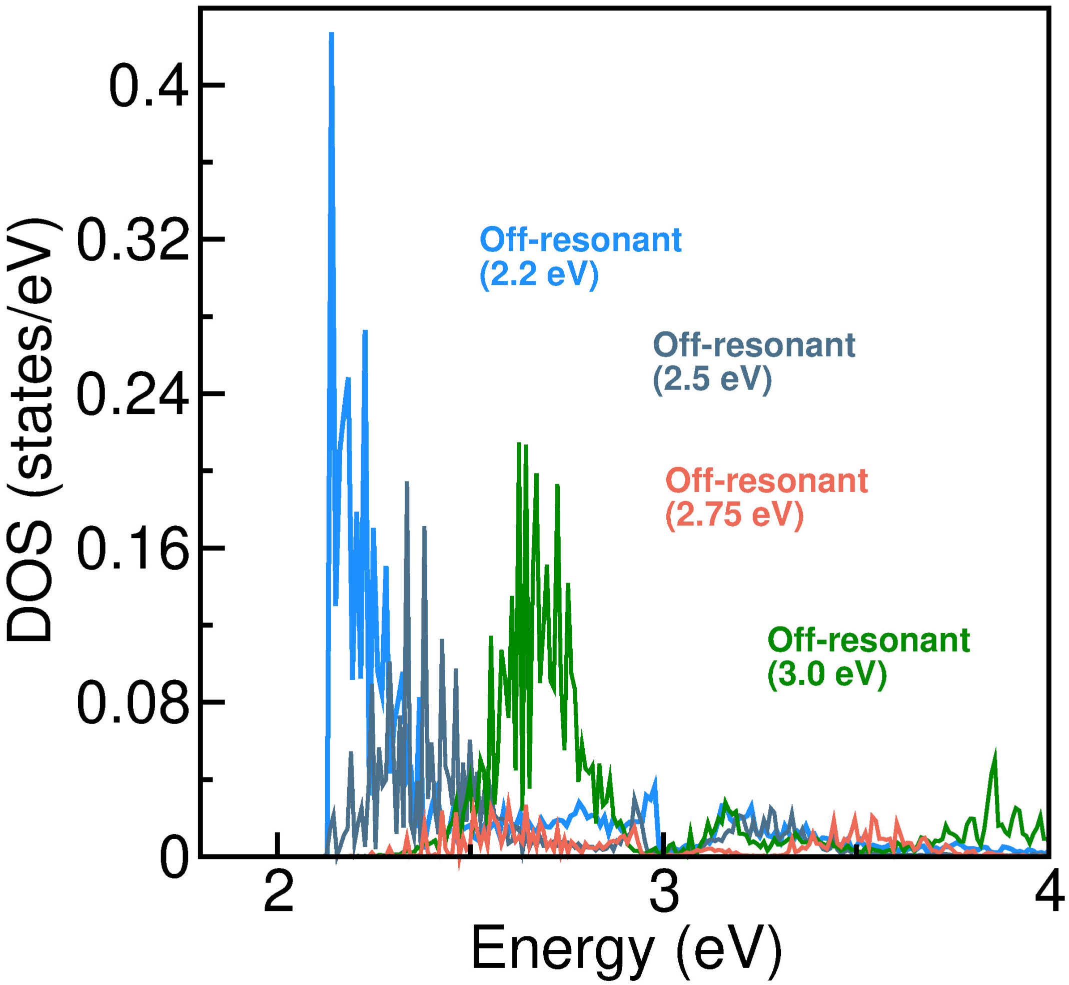

Figure 2: The excited density of occupied states for ML-WSe_2 which is off-resonantly pumped with pulses of various frequencies stated in the legend.

References

(1) Dewhurst, J. K.; Sharma, S.; et al. Jan. 14 **2018**; `elk.sourceforge.net`.

(2) Schutte, W.; De Boer, J.; Jellinek, F. Crystal structures of tungsten disulfide and diselenide. *Journal of Solid State Chemistry* **1987**, *70*, 207–209.

(3) Dewhurst, J. K.; Krieger, K.; Sharma, S.; Gross, E. K. U. An efficient algorithm for time propagation as applied to linearized augmented plane wave method. *Computer Physics Communications* **2016**, *209*, 92–95.

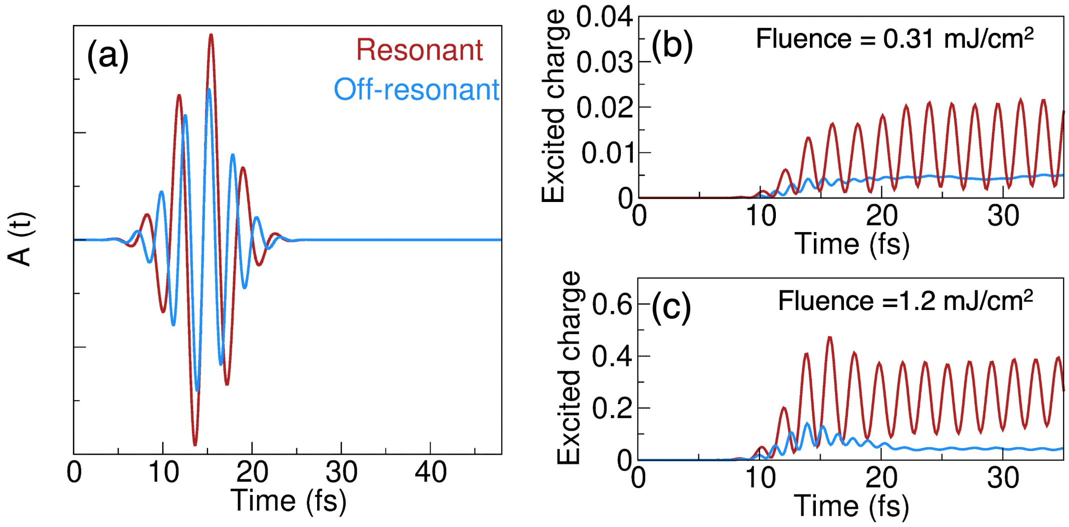

Figure 3: *Free carrier dynamics upon pumping with pulses of short duration of 8 fs;* (a) The vector potential (A(t)) of resonant (red) and off-resonant (blue) pump pulse. Excited charge upon pumping with these pulses while keeping the incident fluence fixed at (b) 0.31 mJ/cm^2 and (c) 1.20 mJ/cm^2. The charge excited by low fluence pulses is almost the same for the resonant and off-resonant case. However, a significant difference can be observed between the excited charge resulting from the higher fluence pulses.

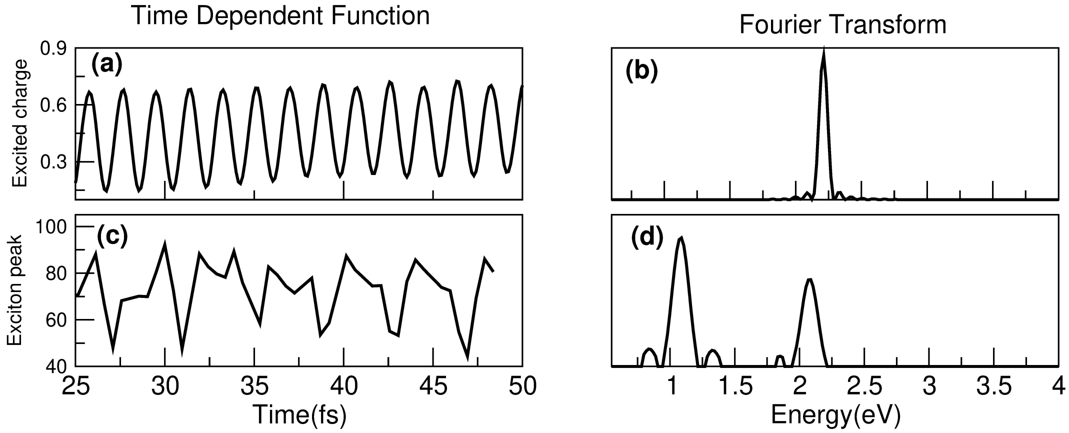

Figure 4: *Fourier analysis of the oscillations;* time dependent oscillations in (a) excited charge and (c) exciton peak height upon pumping with a pulse of short duration of 6 fs. Fourier analysis of these oscillations for (b) excited charge and (d) excitonic peak height. Excited charge oscillations are dominated by the characteristic frequency of the vector field of the pump pulse $\mathbf{A}_{\text{ext}}$. For excitonic response two frequencies contribute, and these correspond to the central frequency of $\mathbf{A}_{\text{ext}}$ and $\mathbf{A}_{\text{xc}}$.

(4) Sharma, S.; Gill, D.; Krishna, J.; Dewhurst, J.; Shallcross, S. Direct coupling of light to valley current. *Nature Communications* **2024**, *15*, 7579.

(5) Pellegrini, C.; Sharma, S.; Dewhurst, J. K.; Sanna, A. Ab initio study of ultrafast de-

magnetization of elementary ferromagnets by terahertz versus optical pulses. *Phys. Rev. B* **2022**, *105*, 134425.

(6) Zhang, C.; Chen, Y.; Johnson, A.; Li, M.-Y.; Li, L.-J.; Mende, P. C.; Feenstra, R. M.; Shih, C.-K. Probing critical point energies of transition metal dichalcogenides: surprising indirect gap of single layer WSe2. *Nano letters* **2015**, *15*, 6494–6500.
